# The complete chloroplast genome sequence of *Pholidota articulata* (Orchidaceae), a rarely medicinal orchid

**DOI:** 10.1080/23802359.2020.1840929

**Published:** 2020-12-24

**Authors:** Chu-Ran Li, Yan Luo, Ping Zhao, Lu Li

**Affiliations:** a Department of Biodiversity Conservation, Southwest Forestry University, Kunming, China; b Department of Forestry, Southwest Forestry University, Kunming, China; c Department of Gardening and Horticulture, Xishuangbanna Tropical Botanical Garden, Chinese Academy of Sciences, Mengla, China; d Key Laboratory of State Forestry and Grassland Administration on Highly-Efficient Utilization of Forestry Biomass Resources in Southwest China, Southwest Forestry University, Kunming, China

**Keywords:** Chloroplast genome, Orchidaceae; *Pholidota articulata*, phylogenetic analysis

## Abstract

*Pholidota* Lindl. ex Hook. was placed in tribe Arethuseae Lindl. (Epidendroideae, Orchidaceae), while its generic relationship has been unclear. Since the plastid genome could play a key role in plant systematics, the complete chloroplast (cp) genome of *P. articulata* was reported in this paper. The cp genome was 160,114 bp in length with four typical quadripartite structures, which was consisted of a large single copy (LSC) region of 87,756 bp, a small single copy (SSC) region of 18,872 bp, and two inverted repeats (IR) of 26,734 bp. In addition, the cp genome encoded 132 genes in total, of which were 113 unique genes, including 79 protein-coding genes, 30 tRNAs, and 4 rRNAs. The phylogenetic analysis indicated that *P. articulata* was closely clustered with other two species of *Pholidota* and that they appeared to be related to *Pleione* in Arethuseae Lindl.

The genus *Pholidota* Lindl.ex Hook. was a small genus comprised about 30 species in Orchidaceae, which was placed in trib. Arethuseae of subfam. Epidendroideae. However, this genus was not monophyly and its generic relationship was unclear (Chase et al. 2015; Li et al. 2016). *Pholidota articulata* Lindl. was an epiphytic orchid which was characterized by the stmelike pseudobulbs and a pendulous, rachis racemose composed of 10 or more greenish white flowers. It was distributed in Southeastern Asia, and occurred in Southern China (Chen et al. 2009), where it was used as a traditionally medicinal resource (Jalal et al. 2009). Since there were few data on *Pholidota*, the chloroplast genome of *P. articulata* was reported for a better understanding of its systematic problem.

Fresh sample of *P. articulata* was harvested from the Wild Orchid Conservation Center of Yunnan Fengchunfang Biotechnology Co., Ltd., which was located in Fumin County, Yunnan Province, China (N25°20′19″, E102°27′26″). The voucher specimen was deposited at the Herbarium of Southwest Forestry University with a specimen code (Lilu 20180009). Total genomic DNA was extracted from fresh leaves using the modified CTAB procedure (Doyle and Doyle 1987) and followed by 150 bp paired-end (PE) sequencing on Illumina Hiseq 2500 platform (Illumina, San Diego, CA) at Shanghai Personal Biotechnology Co., Ltd. The complete chloroplast genome of *P. articulata* was assembled from the clean reads by the GetOrganelle pipe-line (Jin et al. 2018) with the chloroplast genome of *Pholidota imbricata* Lindl. (GenBank MN398392) as reference sequence, and then annotated using the Geneious Prime version 2020.1.2 (Kearse et al. 2012). The annotated chloroplast genome sequence was submitted to GenBank with an accession number MT712149.

The complete cp genome sequence of *P. articulata* was 160,114 bp in length with four typical quadripartite structures, which was contained a large single-copy region (LSC, 87,756 bp), a small single-copy region (SSC, 18,872 bp), and two inverted repeat regions (IR, 26,743 bp). Besides, the cp genome encoded 132 genes, of which were 113 unique genes, including 79 protein-coding genes, 30 tRNAs, and 4 rRNAs. The overall GC content of the whole plastid genome was 37.4%, whereas the corresponding values of the LSC, SSC, and IR regions were 35.3%, 30.5%, and 43.3%, respectively. The structure of CP genome was consistent with those of *P. imbricata* (GenBank, MN398392).

A phylogenetic tree was conducted based on 79 protein-coding genes of 14 orchid species involved to confirm the systematic position of *P. articulate* (Figure 1). These taxa were selected referred to the updated classification of Orchidaceae (Chase et al. 2015; Li et al. 2016), including 4 species from Collabieae Pfitzer and Malaxideae Lindl., 8 from Arethuseae Lindl. as ingroup, together with 2 from Cypripedioideae as outgroup. All these sequence data were downloaded from NCBI GenBank. A maximum-likelihood (ML) analysis was performed by using the CIPRES Science Gateway web server (RAxML-HPC2 on XSEDE 8.2.10) with 1000 bootstrap replicates and settings as described by Stamatakis et al. (2008). The phylogenetic analysis indicated that *P. articulata* was clustered with other two species of *Pholidota* and that they appeared to be related with *Pleione* in Arethuseae.

**Figure 1.**
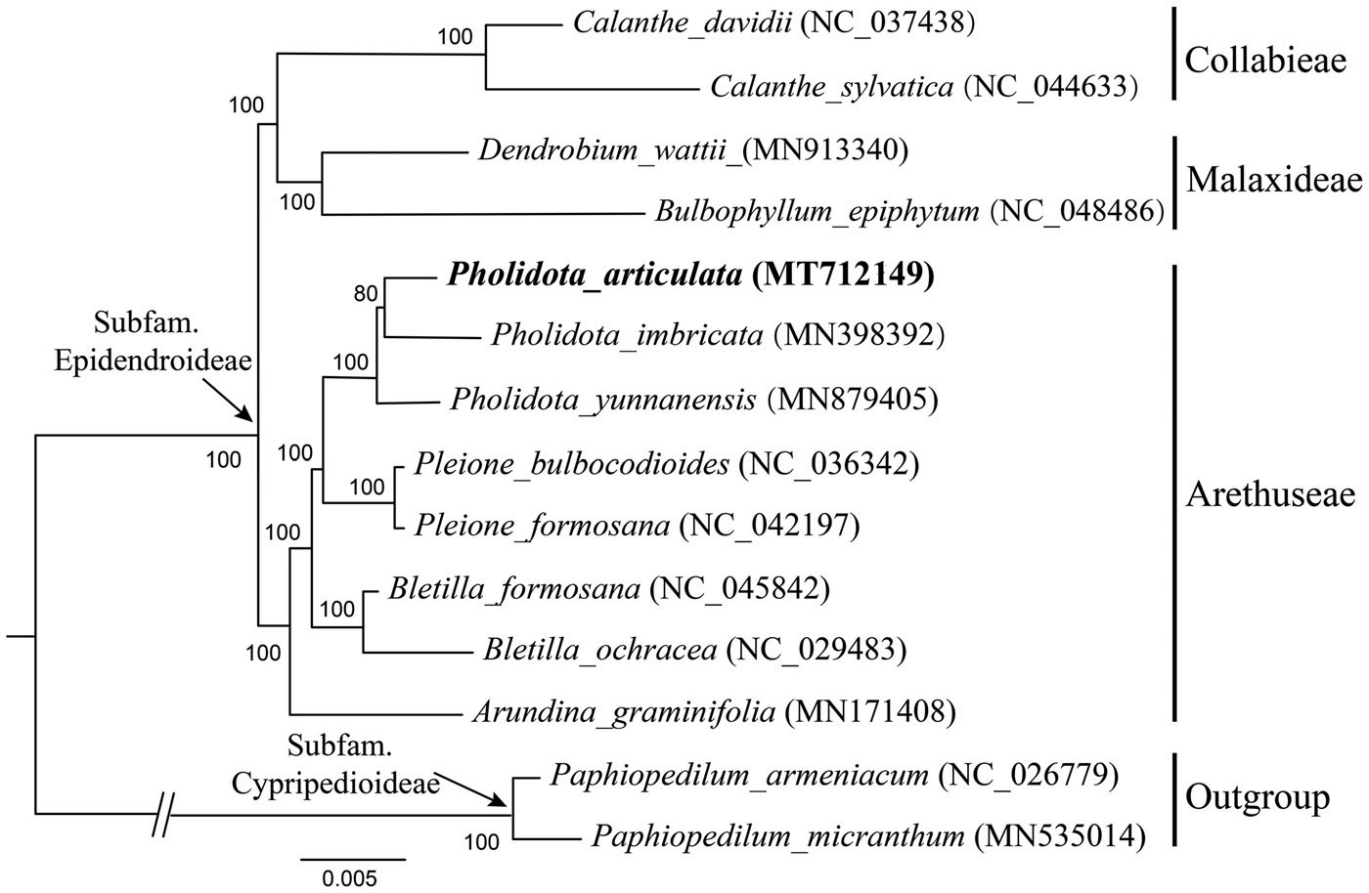
A phylogenetic tree was constructed based on 79 protein-coding genes of 14 orchid species involved. Numbers near the branch indicating the bootstrap values.

## Data Availability

The genome sequence data that support the findings of this study are openly available in GenBank of NCBI at https://www.ncbi.nlm.nih.gov under the accession No. MT712149. The raw sequence data used in this research were deposited successfully with registered numbers of associated BioProject, SRA, and Bio-Sample: PRJNA664054, SRR12669922, and SAMN16197462, respectively.
